# IL-23 and IL-27 Levels in Serum are Associated with the Process and the Recovery of Guillain-Barré Syndrome

**DOI:** 10.1038/s41598-018-21025-5

**Published:** 2018-02-12

**Authors:** Jing Peng, Hui Zhang, Peidong Liu, Min Chen, Bing Xue, Rui Wang, Jifei Shou, Juanfeng Qian, Zhikang Zhao, Yanmeng Xing, Hongbo Liu

**Affiliations:** 1 Department of Neurology, The First Affiliated Hospital of Zhengzhou University, Zhengzhou, Henan P.R. China; 2 Department of Neurology, Beijing Xuanwu Hospital, Affiliated to Capital Medical University, Beijing, P.R. China; 3 Department of Neurosurgery, Tianjin Medical University General Hospital, Tianjin, P.R. China

## Abstract

IL-23 and IL-27 are believed to be involved in the pathogenesis of Guillain-Barré syndrome (GBS). However, changes in these cytokines during the dynamic pathological and recovery processes of GBS are not well described. In the present study, plasma was collected from 83 patients with various stages of GBS, 70 patients with central nervous system demyelinating diseases,70 patients with other neurological diseases (OND) and 70 age- and sex-matched healthy volunteers. Serum levels of IL-23, IL-27, and *Campylobacter jejuni* (CJ) IgM were assessed using enzyme linked immunosorbent assay (ELISA). We found that serum IL-23 levels of patients during the acute phase of GBS were significantly higher followed by a decreasing trend during the recovery phase of the disease. Serum IL-27 levels significantly increased during the acute phase of GBS, and gradually increased during the recovery phase. Interestingly, both the severity and subtype of GBS were closely associated with the two cytokines. IL-23 levels were positively correlated with IL-27 levels, prognosis, and other clinical parameters. Our findings confirm that IL-23 may show pro-inflammatory effects, especially at the early stage of GBS. IL-27 appears to have a dual role in GBS, with initial pro-inflammatory effects, followed by anti-inflammatory properties during recovery.

## Introduction

Guillain-Barré syndrome (GBS) is an autoimmune-mediated neurological disorder affecting peripheral nerves. It is characterised by weakness of limbs and areflexia, and progresses for up to four weeks^1^. It is the most common cause of neuromuscular paralysis. The worldwide incidence of GBS is approximately 1 case per 100,000 people^2^. Commonly thought to have a relatively positive prognosis, in fact up to 20% of patients remain severely disabled throughout their lives, and approximately 5–10% die despite receiving immunotherapy^2,3^.

Divergent but dominant pathologic mechanisms underlie nerve demyelination in GBS, mediated by T cells and macrophages^4^. In the axonal and Miller Fisher variants of the disease, gangliosides (GM1, GD1a, GQ1b) share common antigenic epitopes with bacterial and viral antigens, and are targeted by immunoglobulins^5^. *Campylobacter jejuni* (CJ) infection is associated with the axonal variant in Asia, and affected patients commonly experience more rapid deterioration^6–8^. Many other antecedent infectious agents have been recognized, most recently including the Zika virus^9^.

Cytokines are essential elements in the regulation of inflammatory responses. Pro-inflammatory cytokines, such as TNF-α, IFN-γ, and interleukins (IL)-1β, -6, -12, -17, -18, and -23, as well as anti-inflammatory TGF-β, IL-4 and -10, play various roles in the pathogenesis of GBS^10,11^. A novel IL-12 family member, IL-23, is mainly secreted by activated macrophages and dendritic cells in peripheral tissues including the skin, intestinal mucosa, and lungs^12^. IL-23 is a disulphide-linked complex that shares the p40 subunit with IL-12 and has a different p19 subunit^13^. Several studies have indicated that increased amounts of IL-23 may be associated with some autoimmune diseases, such as multiple sclerosis (MS), rheumatoid arthritis (RA), psoriasis, and inflammatory bowel disease(IBD)^14^. On the other hand, sequential RNA expression of IL-23 p19 is found to be upregulated prior to the onset of the first clinical symptoms in the sciatic nerves of rats with experimental autoimmune neuritis (EAN; an animal model of GBS)^15^. This indicates that IL-23 may be critically involved in the pathogenesis of various immune–mediated disorders.

IL-27 is a newly recognized member of the IL-12 family, and is secreted by activated antigen-presenting cells during antigen presentation to naive T cells^16^. IL-27 exerts both pro-inflammatory and anti-inflammatory effects^17^. Previous studies demonstrated that IL-27 may cause the naïve CD4+ T cells to proliferate and synergized with IL-12 to produce high levels of IFN-γ from activated naïve T cells, setting the stage for commitment to a Th1 phenotype. Studies using IL-27 receptor deficient mice show that the mice were hypersusceptible to developing experimental autoimmune encephalomyelitis (EAE; an animal model of MS), and central nervous system inflammation was more severe^18^. Recent research demonstrated that IL-27 levels are increased in the cerebral spinal fluid (CSF), but not the sera, of relapsing remitting multiple sclerosis (RRMS) patients compared to healthy donors, and that IL-27 is expressed by astrocytes in active MS plaques^19^. Together, this supports the hypothesis that IL-27 may suppress the effector phase of immune-mediated disorders. Conversely, another recent study found that IL-27 might be pathogenic in GBS^20^.

Although IL-23 and IL-27 have been studied in various autoimmune diseases and EAN/EAE models, how they interact with each other, and changes that take place during the dynamic process of progression and subsequent recovery of GBS, are not well described. Little research exists into which of these interleukins may play a role in the acute stage of disease onset, or may promote recovery in the later phase. Moreover, whether there are associations between these two cytokines and prognosis and other clinical parameters in GBS is under-explored. Therefore, we performed a preliminary examination of serum levels of IL-23 and IL-27 during the acute and recovery phases of GBS, to identify potential roles of these cytokines in a longitudinal study.

## Results

### Clinical features of individuals

The GBS group consisted of 83 patients with a mean age of 33.9 ± 11.1 (51 males and 32 females, ranging from 10 to 62 years old). The average ages of central nervous system demyelinating diseases group, other neurological diseases (OND) group, and healthy control (HC)group were 37.8 ± 14.04 (38 females and 32 males, ranging from 15 to 70 years old), 37.27 ± 11.63 (41 males and 29 females, ranging from 16 to 58 years old), and 35.23 ± 10.70 (43 males and 27 females, ranging from 10 to 55 years old), respectively. Demographic and clinical features of patients with GBS are summarized in Table 1.

**Table 1:** The demographic characteristic of subjects.

	electrophysiology		GDSs (onset)		CJ IgM	
	Demyelination type	Axonal type	1–3	4–6	positive	negative
number	47	36	49	34	53	30
Age(y)	33.5 ± 9.9	34.4 ± 12.5	33.8 ± 12.4	34.1 ± 9.0	36.3 ± 11.7	29.7 ± 8.5^**^
Gender (M/F)	26/21	25/11	29/20	22/12	31/22	20/10
Precedent Infection	9/38	10/26	12/37	9/25	22/31	4/26^**^
GDSs at 2 weeks prior to onset	3(2,3)	3(2,4)	2(2,3)	4(3,4)^***^	3(2,3.5)	3(2,3)
Erasmus GBS outcome score	3(2,4)	3(2.625,4)	3(2,3)	4(3,5)^***^	3(2.25,4)	3(2.375,3)
WBC in CSF (10^6^/l)	2(2,4)	2(0.25,3.75)	2(2,4)	2(0,2.25)	2(0.5,4)	2(1.5,2)
Protein in CSF (mg/l)	835(550,1140)	976(569.1,1485.5)	843.4(546.45,1273.05)	890(567.2,1232.75)	950(445.1,1223.5)	871(635.075,1247.475)

Data are expressed as mean ± stand deviation or median (P25, P75). Student’s t-tests, Pearson’s chi-squared tests and d Mann-Whitney U tests were used for statistical analysis. (**<0.01, ***p < 0.001).

Results revealed that age and precedent infection rate in the CJ IgM (+) group were significantly higher (p = 0.009; χ^2^ = 7.070, p = 0.008). The GBS disability scale scores (GDSs) at 2 weeks prior to onset and the Erasmus GBS outcome scores (EGOS) were higher in the severe type compared to the mild type of GBS (p < 0.001; p < 0.001). No statistical significance regarding the age, precedent infection rate, white blood cells (WBC) in CSF and protein content in CSF was found in other subtypes of GBS. In the axonal subtype patients, CJ IgM (+) frequencies were significantly higher than in the demyelination subtype patients (χ^2^ = 5.339, P = 0.021, OR = 3.08, 95% CI (1.165–8.145)). In the severe type group, axonal subtype frequencies were higher than in the mild type group (χ^2^ = 5.597, P = 0.018, OR = 2.946, 95% CI (1.189–7.299)).

### Levels of IL-23 and IL-27 during the process of GBS

**Figure 1:**
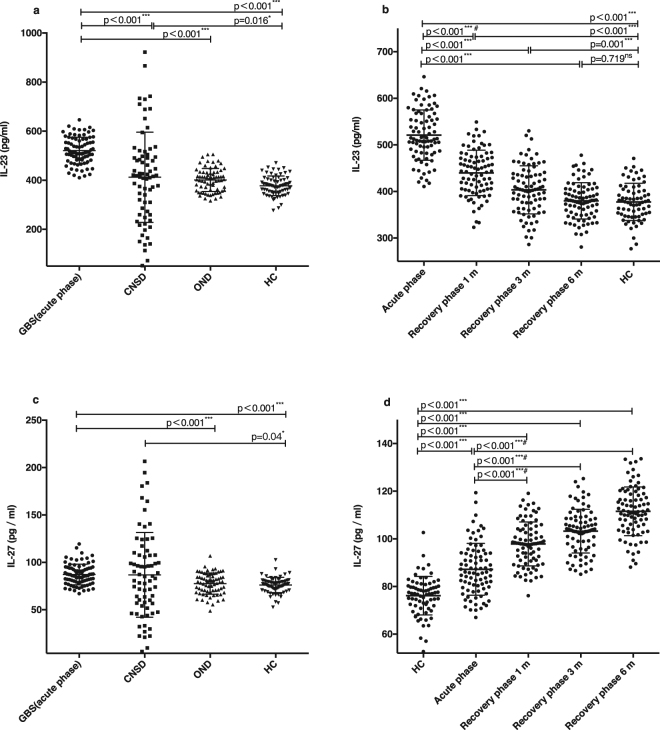
Serum levels of IL-23 and IL-27 in different phases and in different groups. (**a,c**) Comparison of serum IL-23 and -27 concentrations between GBS in acute phase (n = 83) and central nervous system demyelinating diseases (n = 70), other neurological diseases (n = 70), and healthy controls (n = 70) using Kruskal–Wallis. Multiple comparison performed Bonferroni assay. (**b,d**) Comparison of serum IL-23 and -27 concentrations between acute phase and 1 month, 3 months, and 6 months from disease onset using paired t-tests, and between HC group and GBS using Student’s t-tests. (*p < 0.05, **p <0.01, ***p < 0.001, ns indicates not significant, ^#^indicates a non-parametric alternative). CNSD, central nervous system demyelinating diseases; OND, other neurological diseases; HC, health controls; Recovery phase 1 m, after 1 month from disease onset; Recovery phase 3 m, after 3 months from disease onset; Recovery phase 6 m, after 6 months from disease onset.

The mean serum levels of IL-23 and -27 were elevated during the acute phase of GBS when compared to the central nervous system demyelinating diseases group, OND group, and HC group (p < 0.001; p < 0.001; Fig. 1a,c). Meanwhile, levels of IL-23 during the recovery phase showed a decreasing trend, although remained higher than those in the HC group (Fig. 1b). IL-27 concentrations gradually rose (Fig. 1d). Serum IL-23 concentrations during the acute phase were significantly higher than those present after 1 month, 3 months, and 6 months from onset (p < 0.001; p < 0.001; p < 0.001; Fig. 1b). Serum IL-27 concentrations during the acute phase were significantly lower compared to any of the recovery phases (p < 0.001; p < 0.001; p < 0.001; Fig. 1d). At 6 months from GBS onset, serum IL-23 concentrations were not significantly higher than those of the HC group (p = 0.719; Fig. 1b).

### Levels of IL-23 and IL-27 in different GBS subtypes

**Figure 2:**
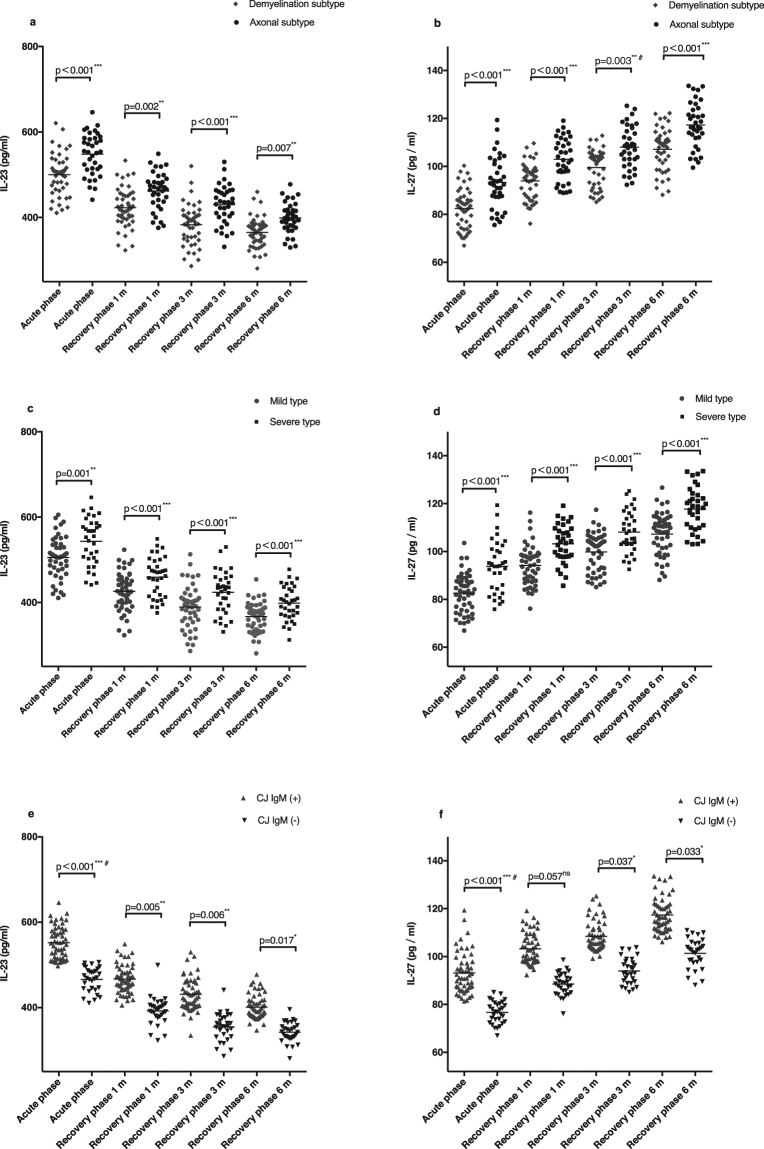
Serum levels of IL-23 or IL-27 in different phases and in different subtypes in GBS using Student’s t-tests (for normally distributed data), or Mann–Whitney U tests (for non-normally distributed data). (**a**) Comparison of serum IL-23 concentrations between axonal subtype (n = 36) and demyelination subtype (n = 47). (**b**) Comparison of serum IL-27 concentrations between axonal subtype (n = 36) and demyelination subtype (n = 47). (**c**) Comparison of serum IL-23 concentrations between wild type (n = 49) and severe type (n = 34). (**d**) Comparison of serum IL-27 concentrations between wild type (n = 49) and severe type (n = 34). (**e**) Comparison of serum IL-23 concentrations between CJ IgM (+) group (n = 53) and CJ IgM (−) group (n = 30). (**f**) Comparison of serum IL-27 concentrations between CJ IgM (+) group (n = 53) and CJ IgM (−) group (n = 30). (*p < 0.05, **p < 0.01, ***p < 0.001, ns indicates not significant, ^#^indicates a non-parametric alternative). Recovery phase 1 m, after 1 month from disease onset; Recovery phase 3 m, after 3 months from disease onset; Recovery phase 6 m, after 6 months from disease onset.

Serum IL-23 and -27 concentrations in patients with axonal subtype GBS were significantly higher than those with demyelination subtype in different phases (Fig. 2a,b). Similar results were observed in the severe group and CJ IgM (+) group when compared with the mild group and CJ IgM (−) group, respectively (Fig. 2c–f). With the recovery of GBS (at 6-month follow-ups) concentrations of IL-23 were decreased and IL-27 were increased when compared with the acute phase in different GBS subtypes (Fig. 2a–f).

### Correlation between serum levels of IL-23 and IL-27

**Figure 3:**
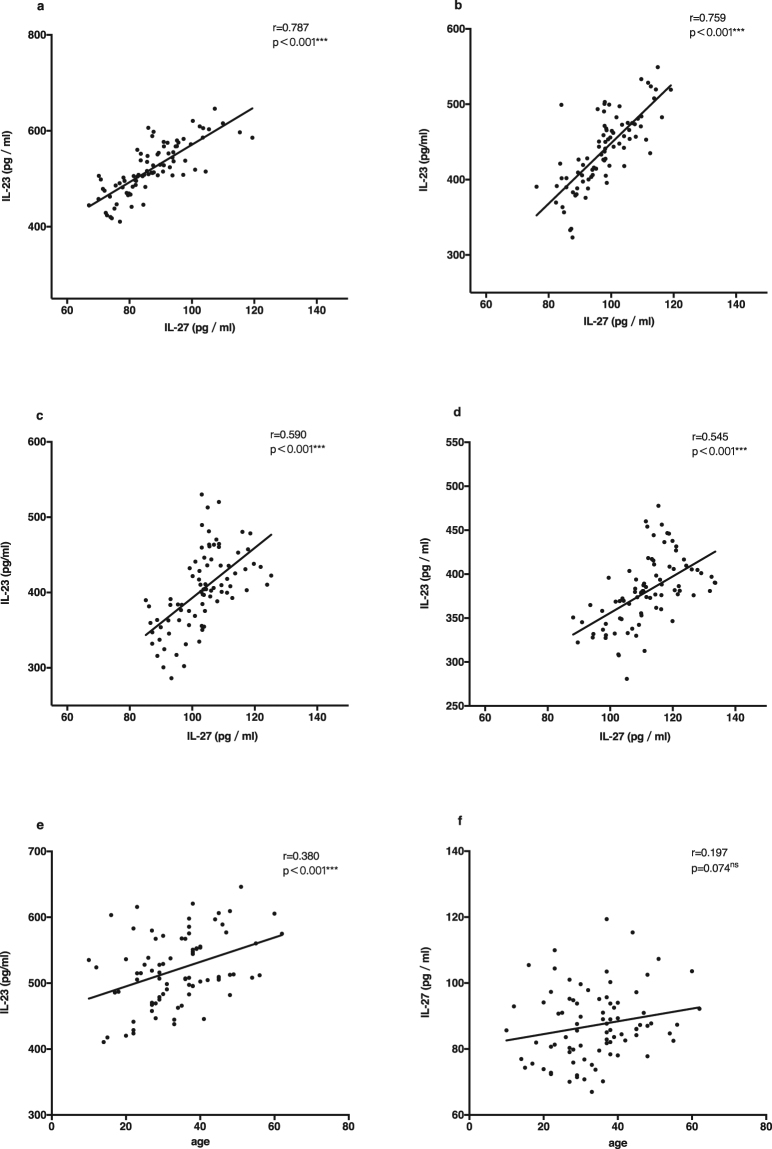
The correlation between levels of IL-23 and levels of IL-27, and between the two cytokines and the age of onset using Pearson’s correlation coefficient. (**a**) The correlation between serum levels of IL-23 and IL-27 in acute phase (n = 83). (**b**) The correlation between serum levels of IL-23 and IL-27 after 1 month from disease onset (n = 83). (**c**) The correlation between serum levels of IL-23 and IL-27 after 3 months from disease onset (n = 83). (**d**) The correlation between serum levels of IL-23 and IL-27 after 6 months from disease onset (n = 83). (**e**) The correlation between serum levels of IL-23 in acute phase and the age of onset in GBS (n = 83). (**f**) The correlation between serum levels of IL-27 in acute phase and the age of onset in GBS (n = 83) (***p < 0.001, ns indicates not significant).

**Figure 4:**
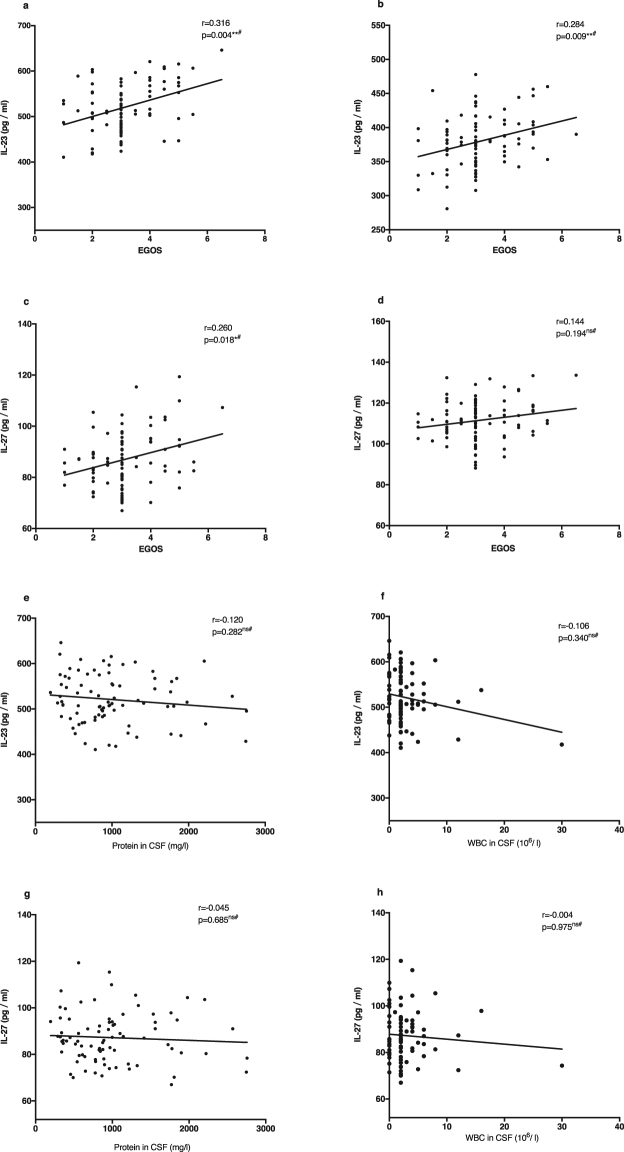
The correlation between serum levels of IL-23, -27 and clinical parameters in GBS using Spearman’s correlation coefficient. (**a**) The correlation between serum levels of IL-23 and EGOS in acute phase (n = 83). (**b**) The correlation between serum levels of IL-23 and EGOS in recovery phase after 6 months from disease onset (n = 83). (**c**) The correlation between serum levels of IL-27 and EGOS in acute phase (n = 83). (**d**) The correlation between serum levels of IL-27 and EGOS in recovery phase after 6 months from disease onset (n = 83). (**e**) The correlation between serum levels of IL-23 and protein in CSF in acute phase (n = 83). (**f**) The correlation between serum levels of IL-23 and WBC in CSF in acute phase (n = 83). (**g**) The correlation between serum levels of IL-27 and protein in CSF in acute phase (n = 83). (**h**) The correlation between serum levels of IL-27 and WBC in CSF in acute phase (n = 83) (*p < 0.05, **p < 0.01, ns indicates not significant, ^#^indicates a non-parametric alternative).

In order to explore the individual roles and potential interactions between IL-23 and IL-27, we performed Pearson correlation analyses. We found that serum IL-23 levels were positively correlated with IL-27 concentrations during the acute and recovery phases (r = 0.787, p < 0.001; r = 0.759, p < 0.001; r = 0.590, p < 0.001; r = 0.545, p < 0.001), a relationship which showed a decreasing trend during disease recovery (Fig. 3a–d). IL-23 concentration was positively correlated with the age of onset in GBS patients (r = 0.380, p < 0.001; Fig. 3e). There was no correlation between IL-27 and age of GBS onset (p = 0.074; Fig. 3f). IL-23 levels at onset and at the 6-month follow-up were positively correlated with EGOS (r = 0.316, p = 0.004; r = 0.284, p = 0.009; Fig. 4a,b), but IL-27 levels at onset were positively correlated with EGOS (r = 0.260, p = 0.018; Fig. 4c,d). There was no correlation between either of the two cytokines and WBC or protein content in the CSF (p = 0.282; p = 0.340; p = 0.685; p = 0.975; Fig. 4e–h).

## Discussion

Cytokine interactions have been reported to be involved in the pathogenesis of GBS. As a newly identified heterodimeric pro-inflammatory cytokine comprising the same p40 subunit and different p19 subunit as IL-12, IL-23 is believed to selectively induce the proliferation of memory T cells^21^. Here, we found that the IL-23 levels in the serum of GBS patients distinctly increased during the acute phase of GBS compared to other groups, and decreased during the recovery phase at 1, 3 and 6 months following disease onset. This suggests that that IL-23 may play a pathogenic pro-inflammatory role in GBS, especially in the early phase of onset. This finding is consistent with previous work using an animal model of EAN, which showed that the IL-23 p19 RNA is upregulated prior to the onset of first clinical symptoms of GBS, with peak expression levels preceding maximum disease severity, and IL-23 p19 protein is detectable in CSF samples from GBS patients, and IL-23 p19 RNA levels decline significantly during the recovery phase^15^. Recent evidence suggests that IL-23, rather than IL-12, is critically involved in the pathogenesis of various immune-mediated disorders, including EAE^22^. One possible mechanism is that IL-23 may promote the development, expansion, and stabilization of Th17 cells, altering the Th1/Th2 paradigm of the immune response in GBS and MS (as well as corresponding animal models)^23–25^. IL-23 is a principle effector cytokine of the Th17 immune response, promoting rapid recruitment of neutrophils and monocytes through the production of induced chemokines^26^. IL-23p19-deficient mice lack Th17 cells, and prolonged *in vitro* culture of Th17 cells required the addition of IL-23. Further, IL-23 drives a population of T cells that can induce EAE and collagen-induced arthritis^27–29^. Together, these data suggest that IL-23 shows pro-inflammatory effects at the early stage of GBS and promotes the progression of the disease, making it a promising therapeutic target. A randomized, double-blinded placebo-controlled study found that the mean relapse rate of patients with RRMS or secondary progressive MS (SPMS) was significantly lower following treatment with IL-12 and IL-23 monoclonal antibodies^30^. Recent research showed that Th17 cells and their cytokines can be therapeutic targets in GBS, for example by using RORγ T inhibitors or IL-23 antagonists^31,32^.

GBS can be classified into demyelinating and axonal subtypes. According to the new diagnostic classification in 2014, the demyelinating subtype “acute inflammatory demyelinating polyneuropathy” (AIDP)—is characterised by demyelination of neurons. This demyelination is absent in the axonal subtypes “acute motor axonal neuropathy” (AMAN) and “acute motor sensory axonal neuropathy” (AMSAN)^33^. AIDP is common in North America and Europe, while AMAN/AMSAN are more commonly found in China, Japan, and Mexico. Our results suggest that the frequency of the axonal subtypes is higher than the demyelination subtype. During the acute phase, GBS patients with axon damage had higher IL-23 levels than the demyelination group, indicating that IL-23 may play an important role in pro-inflammatory induced axonal impairment during the early stage of GBS, and may mediate more severe inflammatory reactions and oedema, ultimately leading to further peripheral nerve injury and muscle weakness.

We found that serum levels of IL-23 during the acute phase were associated with GBS severity. IL-23 levels were significantly higher in the severe group than in the mild group, and showed a decreasing trend during recovery. Similarly, when IL-12p40-deficient mice are induced with EAN, there is a delay in the onset of clinical symptoms, and the incidence and severity of EAN are significantly reduced compared to wild-type controls. On the other hand, IL-23 levels at GBS onset and 6 months later are correlated with the EGOS. Therefore, we hypothesize that higher levels of IL-23 during the acute phase of GBS can not only be a marker of axonal injury, but also a predictor of GBS severity. This may prove valuable in the prognostic evaluation of GBS patients. We further found that the age of GBS onset was positively correlated with IL-23 concentrations in different phases of the disease, suggesting that inflammation may be more harmful with increasing age, which could also prove useful in disease prognosis.

About two-thirds of GBS cases are triggered by antecedent infectious agents, leading to the hypothesis that GBS is a post-infectious immune-mediated disorder. The most frequently identified infectious agent associated with subsequent development of the GBS is CJ, accounting for up to 30% of infections, according to one meta-analysis^34^. Although both axonal and demyelination subtypes have been associated with CJ infection, it has been shown to be more widely associated with AMAN. In the present study, approximately 64% of GBS patients were CJ IgM (+), further supporting that precedent infection of CJ is often found in GBS. The higher proportion of CJ infections in our patients may be due to the poor sanitary conditions in the Henan province of China, consistent with previous research in northern China^35^. Our results showed that IL-23 serum concentrations were higher in the CJ IgM (+) group compared to the CJ IgM (−) group. Within the axonal subtype subjects, CJ IgM (+) frequencies were significantly higher (χ^2^ = 5.339, P = 0.021, OR = 3.08, 95% CI (1.165–8.145)). In severe type group, axon damage type frequencies were higher (χ^2^ = 5.597, P = 0.018, OR = 2.946, 95% CI (1.189–7.299)). Taken together, this suggests that patients with CJ infection may be three times more likely to develop axonal subtype GBS than non-infected patients, and that axonal damage tends to manifest severe clinical symptoms. Evidence suggest that CJ infection may be related to the occurrence of severe GBS and underlie the high incidence of axonal subtype GBS in Asia^6–8^. Our results further support the role of IL-23 in this process.

Another cytokine belonging to IL-12 family, IL-27, has also been the subject of recent investigations^36^. Studies using infectious and autoimmune inflammatory models have shown that mice lacking the WSX-1 subunit of the IL-27 receptor develop excessive pathological inflammation during both Th1 and Th2 responses^37^. Previous studies showed that IL-27 signaling can activate a Th1-specific transcription factor, resulting in the upregulated expression of IL-12R2 on naive T cells^36^. IL-27 can also limit IL-2 production during Th1 differentiation^38^. Conversely, IL-27 contributes to anti-inflammatory responses including decreased differentiation of Th2 and Th17 cells, and increased differentiation of IL-10-producing regulatory T cells^39^.

Recent research revealed that elevated concentrations of serum IL-27 in the AIDP and AMAN subtypes of GBS, inducing the inflammatory processes^20^. Similarly, our results reveal elevated IL-27 in GBS patients compared with OND patients and HC subjects. IL-27 levels increased gradually during the recovery phase, suggesting it may play an important role by participating in the restoration of nerve damage and relieving the progress of GBS via anti-inflammatory mechanisms. It can be supported by several studies. IL-27 significantly inhibits both non-polarised and IL-23-driven IL-17 production by myelin-reactive T cells, thereby suppressing their encephalitogenicity in an adoptive transfer model of EAE^36^. Progressive MS patients have decreased plasma and mRNA levels of IL-27 with increased frequencies of circulating Th17, plasma and mRNA levels of IL-17^40^. IL-27 also contributes to the pathogenesis or immune-regulation associated with T helper cells in myasthenia gravis (MG)^41^. Chong *et al*. found that over expression of IL-27p28 *in vivo* ameliorates experimental autoimmune uveitis (EAU) and EAE pathology^42^. However, IL-27 concentrations in axonal subtype and severe type GBS were significantly higher than in the demyelination and mild symptom groups. Further, we found that serum IL-23 levels were positively correlated with IL-27 concentrations during the acute and recovery phases, suggesting that IL-27 may have a similar role to IL-23. This may imply that IL-27 plays a pro-inflammatory role in GBS, consistent with previous research in MS^19,43^. The elevation of serum IL-27 concentrations in CJ IgM (+) group also indicates an inflammatory role in antimicrobial function and autoimmunity. IL-27 levels at GBS onset, but not 6 months later, are positively correlated with EGOS, which can help in the early prediction of GBS prognosis. Therefore, further studies should seek to clarify pro- and anti-inflammatory effects of IL-27 at various time points during the course of GBS, with particular attention on distinguishing any differences between distinct disease subtypes.

This study is the first to explore dynamic alterations in IL-23 and IL-27 serum levels during various stages of GBS. Correlations of these interleukin levels to disease subtype, severity, and CJ IgM status were also found. Our findings confirmed the inflammatory effects of IL-23 and the immune regulatory role of IL-27 in GBS, and provide new insight into the regulation of inflammatory responses during the pathogenic and recovery processes of GBS. Limitations of this study include small sample sizes and lack of CSF interleukin levels. We previously reported polymorphisms of CD1A genes associated with GBS^44^. Further experiments should use *in vitro* tests to explore the exact mechanisms underlying the interactions of IL-23 and IL-27 with other cells, cytokines, and genes known to be susceptible to the development of GBS.

## Materials and Methods

### Subjects

Eighty-three patients were recruited from the Department of Neurology at the First Affiliated Hospital of Zhengzhou University, China, between July 2010 and February 2015, and diagnosed according to the 1990 Asbury and Cornblath criteria. Patients were excluded if they thought to have GBS deteriorates again after 8 weeks from onset or when deterioration occurs 3 times or more, that was possibly diagnosed as acute onset chronic inflammatory demyelinating polyneuropathy (CIDP), or if they had a history of autoimmune disease such as MS, IBD, RA, or type I diabetes, if they had a recent infection, or if serum samples were not collected during the acute or recovery phases. The central nervous system demyelinating diseases group was comprised of 50 patients with neuromyelitis optica spectrum disorder(NMOSD) and 20 patients with RRMS, all diagnosed according to the international diagnosis criterion. Seventy patients with other neurological diseases were also included, including 25 patients with transient ischemic attack (TIA), 20 patients with benign paroxysmal positional vertigo (BPPV), 10 patients with hypokalemic periodic paralysis, and 15 patients with idiopathic epilepsy. Seventy age- and sex-matched healthy volunteers without organic diseases were used as the HC group. The experimental protocol was approved by the Ethical Committee of the First Affiliated Hospital of Zhengzhou University. The methods were complied with the ethical guidelines for the medical and health research involving human subjects as established by the National Institutes of Health and the Committee on Human Research at the First Affiliated Hospital of Zhengzhou University. All participants provided written informed consent prior to participation.

### Data and sample collection

Demographic features, prior infection history, CSF parameters, GBS disability scale scores (GDSs), Erasmus GBS outcome score (EGOS)^45^, electrophysiology, and therapy data were collected. All participants of the GBS group received electrophysiology testing at 19.4 ± 4.3 days from disease onset and were divided into ‘demyelination’ or ‘axonal’ subtype. The severity of GBS was scored by the use of GDSs. All patients were divided into two groups; those with scores of 1–3 were placed into the mild group and those with scores of 4–6 were placed into the severe group. Two weeks after inclusion in the study, we used the GDSs to evaluate the severity of disease, and the EGOS score to predict 6 month outcomes.

Blood samples of the GBS patients in the acute phase were collected at 9.5 ± 2.5 days from disease onset, before immunosuppression or chemotherapy. Additional blood samples from the same patients were collected during the recovery phase, at 21.4 ± 3.4 days (“1 month”), 3 months, and 6 months from disease onset. All blood samples were collected in the morning and centrifuged within 1 hour. Serum supernatant was collected and stored in Eppendorf centrifuge tubes at −80 °C until use. All patients were treated with intravenous immunoglobulin or plasma exchange.

### Measurement of IL-23 and IL-27 concentrations

The IL-23 and IL-27 concentrations were measured using commercially available human IL-23 and IL-27 enzyme linked immunosorbent assay (ELISA) kits (R&D systems America). CJ IgM status in GBS patients was measured using a CJ IgM ELISA kit (Creative Diagnostics America), using a double antibody sandwich method according to the manufacturer’s instructions. Absorbance was measured at 450 nm, and serum concentrations of IL-23 and IL-27 were calculated according to standard curves. The limits of detection were 16.3 pg/mL and 4.7 pg/mL, respectively. As for CJ IgM, the optical density (OD) was measured within 60 minutes at 405 nm against a blank substrate blank.

### Statistical Analyses

If the data meets the assumptions of normality (Shapiro-Wilk W tests), the results were expressed as mean ± stand deviation, if not, they were expressed as median (P25, P75). Differences in variables were analysed using analyses of Student’s t-tests and paired t-tests (for normally distributed data), or Kruskal–Wallis and Mann–Whitney U tests (for non-normally distributed data) as appropriate. Differences among qualitative variables were tested with Pearson’s chi-squared test. Correlations were analysed using Pearson’s correlation coefficient or Spearman’s correlation coefficient. A two-sided P-value of < 0.05 was considered statistically significant. Statistical analyses were performed using SPSS software (v. 24.0). All figures were made by GraphPad Prism software (v. 7.0). Our data are available to the public.
